# Effect of APE1 T2197G (Asp148Glu) Polymorphism on APE1, XRCC1, PARP1 and OGG1 Expression in Patients with Colorectal Cancer

**DOI:** 10.3390/ijms151017333

**Published:** 2014-09-29

**Authors:** Juliana C. Santos, Alexandre Funck, Isabelle J. L. Silva-Fernandes, Silvia H. B. Rabenhorst, Carlos A. R. Martinez, Marcelo L. Ribeiro

**Affiliations:** 1 Laboratory of Microbiology and Molecular Biology, Clinical Pharmacology and Gastroenterology Unit, Sao Francisco University Medical School, Bragança Paulista, SP. 12916-900, Brazil; E-Mails: ju_carvalho_s@hotmail.com (J.C.S.); afunck1@gmail.com (A.F.); caomartinez@uol.com.br (C.A.R.M.); 2 Genetics and Molecular Biology, UNICAMP, Universidade Estadual de Campinas, Campinas, SP. 13083-862, Brazil; 3 Department of Pathology and Forensic Medicine, Federal University of Ceará, Fortaleza, CE. 60020-181, Brazil; E-Mails: isabellejoyce@gmail.com (I.J.L.S.-F.); srabenhorst@hotmail.com (S.H.B.R.)

**Keywords:** colorectal cancer, DNA repair, APE1 polymorphism, BER

## Abstract

It has been hypothesized that genetic variation in base excision repair (BER) might modify colorectal adenoma risk. Thus, we evaluated the influence of APE1 T2197G (Asp148Glu) polymorphism on *APE1*, *XRCC1*, *PARP1* and *OGG1* expression in normal and tumor samples from patients with colorectal cancer. The results indicate a downregulation of *OGG1* and an upregulation of *XRCC1* expression in tumor tissue. Regarding the anatomical location of *APE1*, *OGG1* and *PARP-1*, a decrease in gene expression was observed among patients with cancer in the rectum. In patients with or without some degree of tumor invasion, a significant downregulation in *OGG1* was observed in tumor tissue. Interestingly, when taking into account the tumor stage, patients with more advanced grades (III and IV) showed a significant repression for *APE1*, *OGG1* and *PARP-1*. *XRCC1* expression levels were significantly enhanced in tumor samples and were correlated with all clinical and histopathological data. Concerning the polymorphism T2197G, GG genotype carriers exhibited a significantly reduced expression of genes of the BER repair system (*APE1*, *XRCC1* and *PARP1*). In summary, our data show that patients with colorectal cancer present expression changes in several BER genes, suggesting a role for *APE1*, *XRCC1*, *PARP1* and *OGG1* and *APE1* polymorphism in colorectal carcinogenesis.

## 1. Introduction

Colorectal cancer is a major cause of cancer-associated morbidity and mortality worldwide. It is the second most prevalent cancer and affects men and women almost equally. At the time of colorectal cancer diagnosis, the majority of cases are no longer confined to the primary site, and the five-year survival rate of metastatic colorectal cancer is below 10% [1]. Colorectal carcinogenesis of humans is possibly one of the best studied and well-characterized process [2]. This is due in part to the relative ease of detection and removal of pre-cancerous lesions, which allows the identification of the sequential stages of colorectal cancer development. It has been described that the molecular basis of colorectal cancer may involve mutations in several genes, such as *APC* and/or those encoding components of DNA repair mechanisms. The accumulation of multiple genetic mutations leads to a selective growth advantage to the epithelial cells in the colon [3].

The DNA repair machinery plays a key role in maintaining genomic stability by preventing the presence of mutations. The DNA repair enzymes continuously monitor chromosomes to correct possible damage caused by exogenous and endogenous mutagens. Base excision repair (BER) is essential for removing oxidized or chemically-modified bases [4]. Indeed, the BER pathway is the predominant mechanism for the repair of oxidative DNA damage; the oxidized base 8-oxoguanine can pair with both adenine and cytosine with, resulting in G:C to T:A transversions [5]. In addition, BER is also involved in the removal of uracil in DNA, which is formed by a spontaneous cytosine deamination process, resulting in erroneous U:G matching [6]. The colon epithelium is one of the most constantly regenerated tissues in the human body and, thus, is more vulnerable to a variety of mutagenic compounds present in the intestine and/or blood. Lifestyle choices, such as smoking and the consumption of alcohol, red meat and processed foods high in saturated fat, have been described as risk factors for developing colorectal cancer through the generation of oxidative DNA damage [7]. It is believed that failure in BER repair is not related to malignant transformation, but rather contributes to tumor mass growth, allowing a greater vulnerability to the accumulation of DNA damage [8].

Poly(ADP-ribose) polymerase-1 (*PARP-1*) is a nuclear enzyme that is activated by DNA strand breaks and is involved in the BER pathway, as well as in the regulation of transcription and the cell cycle [9]. It has been proposed that *PARP-1* has an important role in DNA repair, thereby contributing to the efficient maintenance of genome integrity [10]. In addition, it has been demonstrated that changes in *PARP-1* levels play an important role in colorectal carcinogenesis [11]. Pre-clinical studies have demonstrated that PARP inhibitors can increase radiosensitivity and chemotherapy synergy in experimental models of colorectal cancer. Moreover, several clinical trials have been initiated with the use of PARP inhibitors in patients with colorectal cancer [12].

*XRCC1* (X-ray repair complementing defective repair in Chinese hamster cells 1) is also an important protein that interacts with human polynucleotide kinase (PNK), DNA polymerase β and DNA ligase III to constitute an effective BER repair pathway. Additionally, *XRCC1* stimulates the activities of DNA kinase and phosphatase PNK at damaged DNA termini and accelerates the overall reaction of the repair system [13]. It has been observed that patients with colorectal cancer have high *XRCC1* expression levels that are related to microsatellite instability. However, no association between gene expression and the clinicopathological characteristics of these patients has been observed [14].

Bearing in mind that BER is responsible for removing damage that is largely generated by DNA oxidation, the gene 8-oxoguanine DNA glycosylase (OGG1) plays an important role in the recognition and excision of 8-oxoguanine bases (8-oxoG) formed by exposure to species reactive oxygen [15]. It has been observed that individuals presenting no or low *OGG1* levels have a higher risk of developing lung and head and neck cancer [16]. In a complementary way, it has been shown that patients with colorectal cancer have low levels of *OGG1* expression in tumor tissue compared with normal tissue [8].

In addition to OGG1, AP-endonuclease (APE1) is essential in the repair of oxidized bases in the BER pathway. APE1 is also an important regulator of the DNA-binding of several transcription factors [17]. The Asp148Glu (T2197G-rs3136820) polymorphism in this gene has been associated with an increased risk of colorectal cancer [18,19]; although this polymorphism is located in the endonuclease domain, it does not reduce its activity. It has been proposed that the relationship between the Asp148Glu polymorphism and colorectal cancer risk may be attributed to a reduced ability of APE1 to communicate with other BER proteins, leading to ineffective DNA repair [20].

In view of the role of the polymorphism in BER enzymes in colorectal carcinogenesis, we evaluated the influence of the *APE1* Asp148Glu polymorphism on *APE1*, *XRCC1*, *PARP1* and *OGG1* expression in normal and tumor samples from patients with colorectal cancer. In this manuscript, we described a role for the BER genes, *APE1*, *XRCC1*, *PARP1* and *OGG1*, in colorectal carcinogenesis.

## 2. Results

### 2.1. Patient Population

Among the cases of colon adenocarcinomas, 47% (23/49) were from male patients and 53% (26/49) from females. The patients’ ages varied from 41 to 87 years, with a mean of 65.8 years (±10.3) (Table 1). There was no difference in the disease prevalence between men and women.

Considering the anatomic location, 35% (17/49) of the tumors were located in the colon, and 65% (32/49) were located in the rectum. In the case of colon tumors, 35% (6/17) were present in male patients and 65% (11/17) in female patients. With regard to tumors located in the rectum, 53% (16/30) were present in male patients and 47% (14/30) in female patients (Table 1).

Regarding the tumor node metastasis (TNM) stage, 61% (30/49) of the cases presented a more advanced stage, III and IV. Only 39% (19/49) of the cases were of Stages I and II, stages with a greater chance of survival (Table 1).

**Table 1:** Clinical, histopathological and genotypic characteristics of the samples. TNM, the tumor node metastasis.

Characteristic		Number of Patients (%)
**Gender**	Male	23/49 (47%)
	Female	26/49 (53%)
**Age**	41–62 years	14/49 (29%)
	>63 years	35/49 (71%)
**Location**	Colon	17 (35%)
	Rectum	32 (65%)
**Invasion**	No	17 (35%)
	Yes	32 (65%)
**TNM Classification**	I and II	19 (39%)
	III and IV	30 (61%)
**Genotype**	TT	13/49 (27%)
	TG	26/49 (53%)
	GG	10/49 (20%)

### 2.2. APE1 Genotyping and Expression

Our data showed that 27% (13/49) of the patients were carriers of the wild-type genotype, TT, 53% (26/49) were TG and 20% (10/49) were GG (Table 1).

There were no differences between genotype distribution and age (Table 2). A genotypic analysis according to the anatomical location of the tumor (colon or rectum) also indicated no differences between the studied groups. However, there was a trend in the prevalence of the TG genotype in the colon. Regarding the tumor stage, our data also showed no variation in genotype distribution; however, there was a trend in the prevalence of the TG genotype in the more advanced stage group (Table 2).

Considering that the *APE1* T2197G polymorphism can influence the activity of its gene product, as well as the formation of BER protein complexes, we evaluated the expression pattern of *APE1*, *OGG1*, *PARP-1* and *XRCC1*. The results presented in Table 3 show that the *OGG1* expression was significantly downregulated in tumors samples when compared to normal tissue, while *XRCC1* expression significantly increase. No differences in *APE1* and *PARP-1* expression were observed. Regarding the anatomical location, for *APE1*, *OGG1* and *PARP-1*, there was a decrease in gene expression among the patients with cancer in the rectum compared with the colon. No differences between the sites were observed for *XRCC1*. *PARP-1* upregulation was observed in the colon tumor samples. Interestingly, taking into account the tumor stage, those patients with a more advanced grade (III and IV) showed a significant repression of *APE1*, *OGG1* and *PARP-1*.

Considering that the *APE1*, *OGG1*, *PARP-1* and *XRCC1* genes belong to the same DNA repair system (BER), we evaluated the influence of the *APE1* T2197G (Asp148Glu) polymorphism on the expression of these genes. The data presented show that, with the exception of *XRCC1*, all genes were downregulated among patients carrying the GG genotype when compared with the TT and TG genotypes (Table 3).

**Table 2:** Genotype distribution according to sample characteristics.

Characteristic		*APE1*		
		TT	TG	GG
**Age**	41–62 years	22%	51%	27%
	>63 years	33%	56%	11%
**Location**	Colon	16%	68%	16%
	Rectum	40%	45%	15%
**Invasion**	No	21%	51%	28%
	Yes	29%	58%	13%
**TNM Classification**	I and II	40%	20%	40%
	III and IV	28%	62%	10%

**Table 3:** Gene expression according to sample characteristics.

Characteristic		*APE1*	*OGG1*	*PARP-1*	*XRCC1*
**Fold induction**	Tumor/normal	0.83 ± 0.13	0.28 ± 0.12 ^‡^	1.35 ± 0.12	2.54 ± 1.06 ^‡^
**Location**	Colon	1.09 ± 0.38	0.31 ± 0.07	1.70 ± 0.31	2.25 ± 1.63
	Rectum	0.48 ± 0.02 *	0.17 ± 0.02 *	0.82 ± 0.06 *	2.37 ± 0.68
**Invasion**	Yes	0.73 ± 0.20	0.24 ± 0.04	1.22 ± 0.49	2.59 ± 0.96
	No	0.71 ± 0.10	0.29 ± 0.09	0.89 ± 0.06	2.47 ± 0.60
**TNM Classification**	I and II	1.31 ± 0.24	0.49 ± 0.12	1.98 ± 0.35	3.18 ± 1.26
	III and IV	0.61 ± 0.09 *	0.16 ± 0.08 *	0.90 ± 0.10 *	2.29 ± 0.50
***APE1* Genotype**	TT	1.24 ± 0.25	3.35 ± 1.43	1.81 ± 0.36	0.45 ± 0.10
	TG	1.02 ± 0.10	3.19 ± 1.01	1.82 ± 0.50	0.43 ± 0.15
	GG	0.39 ± 0.09 ^φφ^	1.72 ± 0.40 ^φ^	0.50 ± 0.10 ^φφ^	0.31 ± 0.09

^‡^ *p* < 0.05 when compared to normal tissue; * *p* < 0.05 when compared between characteristics; ^φ^ *p* < 0.05 and ^φφ^ *p* < 0.01 when compared with wild-type TT.

## 3. Discussion

It is well known that the DNA repair machinery plays a key role in maintaining genomic stability by preventing the existence of mutations. Thus, taking into account the role of BER in colorectal carcinogenesis, the effects of the *APE1* Asp148Glu polymorphism on *APE1*, *XRCC1*, *PARP1* and *OGG1* gene expression were evaluated in patients with colorectal cancer.

Currently, the pathological stage, as established by the International Union Against Cancer [21], is considered the main indicator of the tumor and is very important in therapeutics. The samples included in this study were, in most cases, at a more advanced stage (III and IV), as also previously reported [22,23].

To date, there are 18 SNPs reported in *APE1*, but the most extensively studied is the transversion T2197G (Asp148Glu) [24]. Our data show a higher frequency of the heterozygous genotype TG, followed by the wild-type TT and the homozygous variant GG. A similar distribution was observed in a study conducted in Poland [25], China [26], Turkey [27] and northeastern Brazil [28] in others cancers. The Asp148Glu polymorphism has been associated with an increased risk of colorectal [25], lung [29] and head and neck [30] cancers. It has been suggested that changes in *APE1* might have an impact on endonuclease and DNA-binding activities [31], including the transcriptional regulatory function. Additionally, it has been shown that the presence of the *APE1* Asp148Glu decreases the expression of *APE1*, *XRCC1* and *PARP1 in vitro* [32]. The data presented in this study are consistent with the above study, because the GG genotype affected *APE1* expression, as well as reduced *XRCC1* and *PARP1 in vivo*, which may have an effect on the efficiency of removing the DNA adducts in the host cells.

Moreover, irrespective of this polymorphism, we observed a significant repression of *APE1* in patients with cancer in the rectum and with a more advanced grade (III and IV). However, it has been described that increased levels of 8-oxoGua in leukocytes from patients with adenocarcinoma were related to higher *APE1* mRNA levels [33]. Thus, changes in mRNA levels may occur during the process of carcinogenesis, with the upregulation of most DNA repair gene repair being an early event, following repression at a later stage, as previously demonstrated [34].

Because it has been proposed that *PARP-1* has an important role in DNA repair [10], the expression of this gene was analyzed. Our data showed a downregulation among patients with cancer in the rectum and with a more advanced grade (III and IV). In contrast, an upregulation was observed in tumor samples from the colon. Indeed, this cancer exhibits a wide heterogeneity, and it has been demonstrated that *PARP*-1 expression levels vary from lower to higher levels, depending on the tumor type [35].

In relation to *OGG1*, a significant downregulation was observed in the tumor samples compared to the normal samples, as well as all clinical, histopathological data. Similar data were observed previously [8]. It is well known that the OGG1 enzyme is responsible for catalyzing the excision of 8-oxoGua, which plays an important role in carcinogenesis, because it may cause GC > TA transversions and genomic instability [36]. Thus, the decrease in its activity found in the population studied could have possibly contributed to the development of colorectal cancer.

In contrast to *OGG1*, *XRCC1* showed significantly enhanced expression levels in the tumor samples, as well as in all clinical, histopathological data. Given that *XRCC1* stimulates the kinase and phosphatase activities of PNK DNA at damaged DNA termini and accelerates the overall reaction of the repair system [13], the upregulation observed in this study may be a response to the DNA damage generated during carcinogenesis. In a complementary manner, an induction in *XRCC1* expression has been observed in keratinocytes after UVB radiation [37]. Our data suggest that several changes in gene expression might occur after DNA damage, resulting in a tumor cell profile.

## 4. Experimental Section

### 4.1. Patients

This study was approved by the Ethics Committee in Research of University of São Francisco (Project No. 0235.0.142.000-07). All patients who provided biological material for the research signed a consent form after being informed of all of the experimental stages.

We selected 49 individuals with adenocarcinoma of the colon and upper rectum and who underwent colorectal surgery with curative intent by the same surgical team between January, 2010, and December, 2013. Exclusion criteria were as follows: (1) suspicion of the patient belonging to a family with hereditary colorectal cancer (familial adenomatous polyposis and hereditary non-polypoid colorectal cancer); (2) patients with colorectal cancer associated with inflammatory bowel disease; (3) patients undergoing surgery on an emergency basis; and (4) patients with cancer of the medium and lower rectum undergoing neoadjuvant chemoradiation therapy.

Immediately after removal of the surgical specimen, fragments were removed from normal colonic mucosa at least 10 cm away from the proximal edge of the tumor. Similarly, fragments were collected from the neoplastic mucosa, always obtaining them from the periphery of the tumor. The identified fragments were removed, packaged individually in appropriate containers and immediately cooled to −80 °C until testing.

Epidemiological, clinical and pathological data were obtained during an interview with the patient and by consulting the medical records. Histopathological data, such as tumor subtype, depth of invasion, lymph node and/or metastasis distance and staging, were extracted from the pathological reports present in their medical records. The tumors were staged pathologically using the tumor node metastasis (TNM) system.

### 4.2. RNA Extraction and Real-Time PCR

The biopsies were collected, snap frozen and stored at −80 °C in RNAlater (Qiagen, Valencia, CA, USA). Total RNA was isolated using the RNeasy tissue kit (Qiagen). The purity and yield of total RNA isolated was assessed by measuring the optical density using a NanoDrop 2000 spectrophotometer (Thermo Scientific, Wilmington, DE, USA). Single-stranded cDNA was synthesized from the RNA using the high-capacity cDNA archive kit (Applied Biosystems, Foster City, CA, USA) following the manufacturer’s protocol.

Quantitative PCR was performed with a 7500 real-time PCR system (Applied Biosystems) using threshold cycle numbers determined by the RQ Study software (Applied Biosystems). The reactions were run in triplicate, and the threshold cycle numbers were averaged. The 50-µL reaction mixture was prepared as follows: 25 µL Sybr Green^®^ Quantitative PCR SuperMix-UDG (Invitrogen Life Technologies, Alameda, CA, USA), 10 mM of each primer (Table 4) and 1 µL of cDNA (100 ng). The reaction was cycled with a preliminary UDG treatment for 2 min at 50 °C and denaturation for 2 min at 95 °C, followed by 45 cycles of denaturation at 95 °C for 15 s, annealing at 60 °C for 15 s and primer extension at 72 °C for 15 s. This step was followed by a melting-point analysis of the double-stranded amplicons consisting of 40 cycles of 1 °C decrements (15 s each) beginning at 95 °C. The first derivative of this plot, dF/dT, is the rate of change of fluorescence in the reaction. A significant change in fluorescence occurs at the melting point of the double-stranded PCR products. A plot of dF/dT *versus* temperature displays these changes as distinct peaks.

*APE1*, *XRCC1*, *PARP1* and *OGG1* expression was measured and normalized to the constitutive 18S gene, which showed constant expression in all of the samples tested. The relative expression was calculated according to the formula 2^(−∆∆*C*t)^, and the results are expressed as average gene expression ± SD.

**Table 4:** Primers used for PCR-RFLP and real-time PCR.

Gene	Sequence (5'–3')
*APE1*	CTGCTCTTGGAATGTGGATG
	TTTGGTCTCTTGAAGGCACA
*PARP1*	TAGCTGATGGCATGGTGTTC
	GACGTCCCCAGTGCAGTAAT
*OGG1*	CCTGTGGGGACCTTATGCT
	CCTTTGGAACCCTTTCTGC
*XRCC1*	GTTCCAGCAGTGAGGAGGAT
	GTGGGCTTGGTTTTGGTCT
*18S*	CGCGGTTCTATTTTGTTGGT
	CGGTCCAAGAATTTCACCTC

### 4.3. DNA Extraction and APE1 Asp148Glu Polymorphism

DNA was extracted using a phenol-chloroform method only when the histopathological analysis determined that the tumor specimens consisted mainly (>80%) of tumor cells. The absorbance at 260/280 and 260/230 nm was determined for each sample using a NanoDrop 2000 spectrophotometer.

Single-nucleotide polymorphisms (SNPs) for *APE1* T2197G (rs3136820) were determined using a PCR-restriction fragment length polymorphism (RFLP)-based method.

PCR products were generated using, in each reaction, a total volume of 25 µL containing 10 pM of each primer, 3.6 U Platinum Taq DNA polymerase (Invitrogen), 0.3 mM dNTPs, 2.0 mM MgCl_2_ and 100 ng DNA template.

The reactions proceeded under the following conditions: initial denaturation at 94 °C for 4 min, followed by 35 cycles of 94 °C 45 s, 48.5 °C 45 s and extension at 72 °C for 1 min. An extension period for 5 min followed the final cycle. Negative (water) and positive controls were assayed in each run. The amplified fragments were visualized on 2% agarose gels containing ethidium bromide under UV light. The 164-bp PCR products were digested with *Bfa*I (NEB) following the manufacturer’s protocol. Briefly, in the 2197G allele, the enzyme recognizes the restriction site and generates two fragments of 144 and 20 bp; the restriction site is abolished in the 2197T allele. The fragments were resolved by 8% nondenaturing polyacrylamide gel electrophoresis and silver staining. Randomly selected samples were re-genotyped (10% of the samples).

### 4.4. Statistical Analysis

Statistical analyses were conducted using the SPSS v.12.0 statistical software program (SPSS, Chicago, IL, USA). Statistically significant differences were evaluated by the chi-square test (χ^2^) and Fisher’s exact test. The expression analysis was assessed by an unpaired Student’s *t*-test. A *p* value of <0.05 was considered statistically significant.

## 5. Conclusions

In summary, by combining the genotypic profile of genes that affect the ability of the DNA repair machinery, our findings provide an important contribution to our knowledge of the genesis of colorectal cancer. Furthermore, our data show that patients with colorectal cancer present expression changes in several BER genes, suggesting a role for *APE1*, *XRCC1*, *PARP1* and *OGG1* in colorectal carcinogenesis.
